# Cardioprotection induced in a mouse model of neuropathic pain via anterior nucleus of paraventricular thalamus

**DOI:** 10.1038/s41467-017-00891-z

**Published:** 2017-10-10

**Authors:** Yi-Fen Cheng, Ya-Ting Chang, Wei-Hsin Chen, Hsi-Chien Shih, Yen-Hui Chen, Bai-Chuang Shyu, Chien-Chang Chen

**Affiliations:** 1 Graduate Institute of Life Sciences, National Defense Medical Center, Taipei, 114 Taiwan; 2 Institute of Biomedical Sciences, Academia Sinica, Taipei, 115 Taiwan; 3 International Graduate Program in Molecular Medicine, National Yang-Ming University and Academia Sinica, Taipei, 115 Taiwan

## Abstract

Myocardial infarction is the leading cause of death worldwide. Restoration of blood flow rescues myocardium but also causes ischemia-reperfusion injury. Here, we show that in a mouse model of chronic neuropathic pain, ischemia-reperfusion injury following myocardial infarction is reduced, and this cardioprotection is induced via an anterior nucleus of paraventricular thalamus (PVA)-dependent parasympathetic pathway. Pharmacological inhibition of extracellular signal-regulated kinase activation in the PVA abolishes neuropathic pain-induced cardioprotection, whereas activation of PVA neurons pharmacologically, or optogenetic stimulation, is sufficient to induce cardioprotection. Furthermore, neuropathic injury and optogenetic stimulation of PVA neurons reduce the heart rate. These results suggest that the parasympathetic nerve is responsible for this unexpected cardioprotective effect of chronic neuropathic pain in mice.

## Abstract (web summary)

Various forms of preconditioning can prevent ischemic-reperfusion injury after myocardial infarction. Here, the authors show that in mice, the presence of chronic neuropathic pain can have a cardioprotective effect, and that this is dependent on neural activation in the paraventricular thalamus.

## Introduction

Ischemic heart disease or myocardial ischemia is the leading cause of death worldwide and often responsible for sudden death^1^. The principle intervention is timely thrombolysis or primary coronary angioplasty to restore blood supply into the occluded myocardium. However, reperfusion can also damage cardiomyocytes due to calcium overload, free radical production, and inflammatory cell infiltration. This phenomenon is called ischemia-reperfusion (IR) injury^2^. It is possible to make the heart more resistant to IR injury by pre-exposing the heart to several cycles of short coronary occlusion-reperfusion before the global ischemia, a procedure called ischemic preconditioning (IPC)^3^. Preconditioning of brief IR episodes can also be applied in distant tissues or organs to protect heart from IR injury which is called remote IPC (RIPC)^4–6^.

In addition to ischemic triggers, cardioprotection can also be induced by non-ischemic stimulation. For example, peripheral nociception induced by skin incisions on the abdomen provided cardioprotection and called remote preconditioning of trauma (RPCT) in rodent^7^. Topical application of 0.1% capsaicin cream on the abdomen before IR also reduced infarct size. RPCT required neurogenic signaling involving spinal nerves, sympathetic nerves, and activation of PKCε in the heart^7^. This study demonstrated a beneficial effect of acute nociceptive stimulation against myocardial infarction. Other cardioprotective non-ischemic manipulations include direct peripheral nerve stimulation and noninvasive transcutaneous electrical nerve stimulation^8, 9^.

Prodromal angina, presented as a form of chest pain, can limit infarct size and is speculated as an innate cardioprotection^10–12^. Preinfarction angina is associated with significant cardioprotection (greater than 50% reduction in infarct size) in patients received percutaneous coronary intervention during ST-elevation myocardial infarction^13, 14^. Although preinfarction angina-associated cardioprotection is thought to represent a clinical correlation of IPC, it is possible that angina also induces nociceptive signal pathway to provide cardioprotection. A recent study demonstrated that acute (15 min), delayed (24 h), or chronic (9 days) RIPC elicited similar cardioprotective effects in mice^15^. This study suggests that cardioprotection could be achieved by both acute and delayed or chronic phase of conditioning. It is unclear whether pre-existing chronic pain will also have a similar cardioprotective effect.

A worldwide survey shows that up to 25% of the population is suffering from chronic pain, and up to 8% is under chronic neuropathic pain, especially in elder^16–18^. Ischemic heart disease is also prevalent in the elderly population^19^. Several studies examine the relationship between chronic pain and cardiovascular diseases (CVD) risk focused on the elevated blood pressure or hypertension^20–24^. These studies show that there is a positive relationship between chronic pain and CVD risk. However, it is unclear whether this is a relationship between chronic pain and ischemic heart diseases.

In this study, we aim to determine whether chronic pain can limit IR injury. Using spared nerve injury (SNI) neuropathic pain model, we showed that SNI but not sham operation reduced the infarct size after IR injury in mice. We also showed that the extracellular signal-regulated kinase (ERK) activity and neuronal activity in the anterior nucleus of the paraventricular thalamus (PVA), a brain region located at the rostral portion of paraventricular thalamus (PVT), is required for the SNI-induced cardioprotection. In addition, direct activation of PVA neuron using pharmacological or optogenetic tools without peripheral injury also provided cardioprotection. Activity of the autonomic nervous system is important in the RIPC^25–27^, we treated mice with autonomic nerve blockers and showed that parasympathetic but not sympathetic blocker abolished the SNI-induced cardioprotection. Overall, our results demonstrate that chronic pain induces cardioprotection via a central mechanism involving activation of PVA neurons.

## Results

### Chronic neuropathic pain elicits cardioprotection

**Fig. 1:**
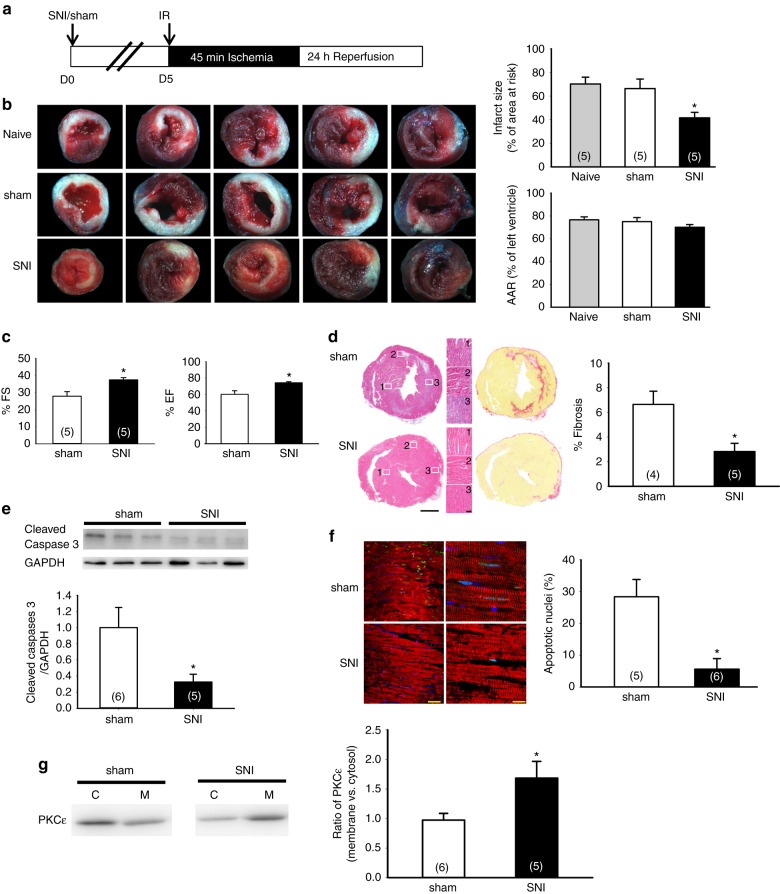
SNI-induced chronic neuropathic pain provides cardioprotection against IR injury. **a** Schematic of experimental design showing the timeline for SNI surgery (D0) and myocardial IR surgery (D5). **b** *Left panel*, representative images of TTC staining showing area of infarct region (*pale color*) and AAR in heart cross-sections from different treatments. Evans blue dye was injected retrogradely from aorta into coronary circulation to delineate the remote area. The infarcted size was determined by TTC (1%) staining. *Right panel*, quantification results of infarct size and AAR. *p < 0.05 SNI vs. sham and naïve groups. **c** Percentage of fractional shortening (% FS) and ejection fraction (% EF) from sham and SNI groups by echocardiography. *p < 0.05 SNI vs. sham. **d** Representative images of H&E staining (*left panel*) and picrosirius red staining (*right panel*) of cardiac sections 4 weeks after IR injury from sham and SNI groups. Areas marked by *white rectangles* were magnified and shown in the *middle panel*. *Scale bar* = 1 mm and 50 μm. Bar graph, quantification results of fibrosis in left ventricles. *p < 0.05 SNI vs. sham. **e** *Upper panel*, immunoblotting of cleaved caspase 3 from left ventricle lysate 24 h after IR in SNI or sham groups. *Lower panel*, quantification results of cleaved caspase 3 immunoblotting. *p < 0.05 SNI vs. sham. **f** Representative images of TUNEL stain in cardiac sections (*red*: α-actinin, *green*: TUNEL signal, *blue*: DAPI). Quantification was conducted by entire slices scanning and TUNEL-positive signals (green fluorescence) were normalized to total nuclei (DAPI, blue fluorescence). Apoptotic activity was assayed by TUNEL stain 24 h after IR injury in SNI or sham groups. *p < 0.05 SNI vs. sham group. *Scale bar* = 50 μm (*left panel*) and 10 μm (*right panel*). **g** Representative immunoblotting and quantification of cytosolic and membrane fraction of PKCε in left ventricular lysate. *p < 0.05 SNI vs. sham group. *Error bars* indicate SEM. Sample numbers are indicated within *parentheses* in all figures. Statistical significance was determined by one-way ANOVA (**b**) or Student’s *t-*test (**c**–**g**)

To investigate whether chronic pain provides cardioprotection, we induced myocardial IR injury in mice 5 days after SNI (Fig. 1a). Left anterior descending coronary artery (LAD) was occluded for 45 min and reperfused for 24 h before examining the degree of myocardium damage. The results from triphenyltetrazolium chloride (TTC) staining clearly exhibited a reduced myocardial infarction (indicated by the pale color region in the transverse section of hearts) in the SNI compared to those in the naïve and sham groups (Fig. 1b). Quantification of TTC staining showed a significant reduction in infarct size in the SNI chronic neuropathic pain group (41.4 ± 4.7%, *n* = 5) compared to naïve (70.1 ± 5.8%, *n* = 5) or sham (66.2 ± 8.1%, *n* = 5) groups. To ensure the difference in the infarct size is not caused by different myocardium injury, we measured the area at risk (AAR) and found there is no difference among these three groups. To examine whether SNI surgery could alter hemodynamic change, we conduced blood pressure and echocardiographic measurement 5 days after SNI or sham surgery. There is no significant difference on the blood pressure between sham and SNI animals 5 days after surgery. The systolic pressure of sham and SNI animals before surgery were 107.2 ± 1.7 (*n* = 5) and 109.5 ± 2.7 mmHg (*n* = 5), respectively. The systolic pressure of sham and SNI animals 5 days after surgery were 108.2 ± 2.7 (*n* = 5) and 115.1 ± 1.9 mmHg (*n* = 5), respectively (Supplementary Fig. 1). There is also no significant difference on the parameters measured by echocardiography between sham and SNI groups 5 days after surgery (Supplementary Fig. 2). These results suggest that SNI-induced cardioprotection is not mediated by hemodynamic alteration induced by SNI or sham surgery. We also measured the serum level of creatine kinase muscle and brain isoenzyme (CKMB) as a cardiac injury marker 24 h after IR injury. CKMB levels were significantly lower in the SNI compared to sham groups, which indicates less cardiac injury in SNI group (Supplementary Fig. 3). Four weeks after IR injury, ejection fraction and fraction shorting measured by echocardiography were significantly higher in SNI compared to sham group (Fig. 1c). Histological examination of the cardiac transverse section 4 weeks after IR injury also showed reduced fibrosis and preserved left ventricular free wall in SNI compared to sham groups (Fig. 1d). Cardiomyocytes apoptosis leads to cell death in IR injury^28^. Therefore, we examined apoptosis at AAR after IR injury by terminal deoxynucleotidyl transferase-mediated dUTP nick end-labeling (TUNEL) assay and detection of cleaved caspase 3. The amount of cleaved caspase 3 was significantly reduced in the SNI group (Fig. 1e). TUNEL assay also showed a significant reduction in cell apoptosis in SNI (5.6 ± 3.3%, *n* = 6) compared to the sham group (28.4 ± 5.4%, *n* = 5) (Fig. 1f). TUNEL-positive cells were co-localized with α-actinin-positive cells, indicating apoptotic cardiomyocytes after IR injury (Fig. 1f). One of the downstream effectors in remote cardioprotection is activation of PKCε; once activated, PKCε is translocated from cytosol to membrane fraction^29^. We analyzed PKCε translocation in hearts isolated from mice with neuropathic pain and the results showed the amount of PKCε increased in membrane fraction in SNI group compared to the sham group (Fig. 1g). These results suggest a remote cardioprotective effect can be induced by SNI-induced chronic neuropathic pain model in mice.

### Attenuation of pain does not inhibit cardioprotection

**Fig. 2:**
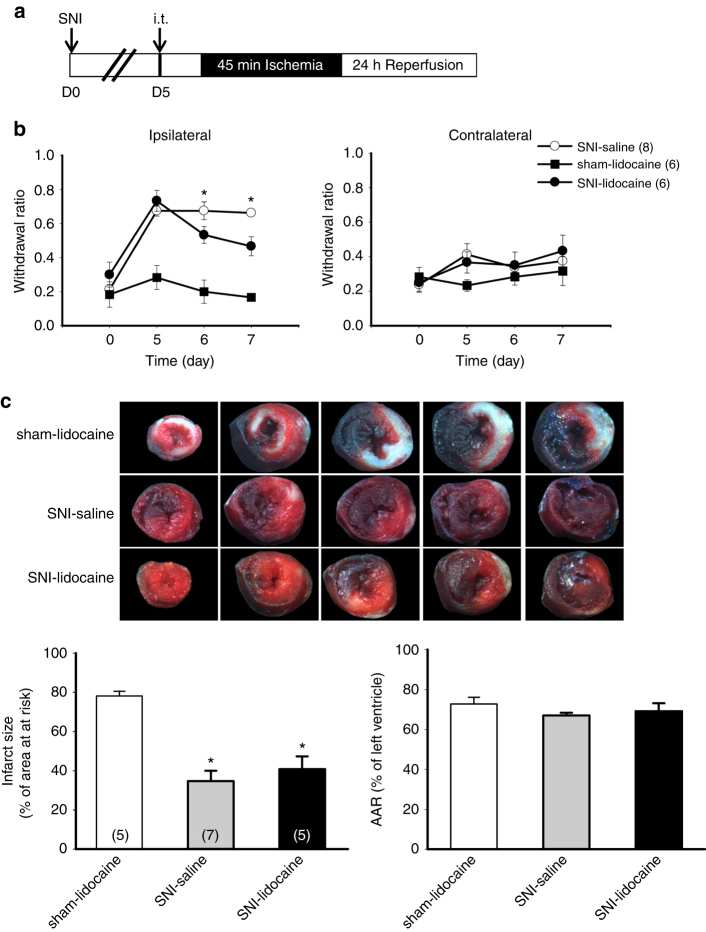
Intrathecal injection of lidocaine reduces mechanical hyperalgesia but not SNI-induced cardioprotection. **a** Schematic of experimental design showing the timeline for SNI surgery (D0), intrathecal infusion (i.t.) of lidocaine (15 mg/kg, in 4 μL saline) (D5), and myocardial IR surgery (D7). **b** Mechanical responses of hind paws in animals received saline or lidocaine infusion at the contralateral side and ipsilateral side. Mechanical responses of hindpaw were measured at day 0 (D0) before SNI surgery, D5 before lidocaine/or saline infusion, D6 and D7 using von Frey filament test. *p < 0.05 vs. saline group. **c** Representative images of TTC staining in heart cross-sections. Quantification results of infarct size and AAR in cardiac sections from saline and lidocaine-infused animals. SNI-induced cardioprotection was not affected even though pain response was reduced. *p < 0.05 vs. sham-lidocaine group. *Error bars* indicate SEM. Statistical significance was determined by two-way RM ANOVA (**b**) or Student’s *t-*test (**c**)

We next asked whether maintenance of chronic pain is required to induce cardioprotection. To answer this, we infused lidocaine (15 mg/kg), a local anesthetic, intrathecally 5 days after SNI (Fig. 2a). Intrathecal infusion of lidocaine has been shown to relieve hyperalgesia in a rat model of chronic neuropathic pain^30^. Mechanical behavior test indicated intrathecal injection of lidocaine attenuated SNI-induced chronic mechanical hypersensitivity in mice (Fig. 2b). Interestingly, relief of mechanical hypersensitivity does not diminish the cardioprotective effect induced by SNI surgery. The infarct size was 34.8 ± 5.3% in the vehicle group (*n* = 7) and 40.8 ± 6.5% in the lidocaine group (*n* = 5), respectively (Fig. 2c). To rule out the effect of lidocaine on cardioprotection independent of neuropathic pain, we also infused lidocaine in sham animals. The results showed that lidocaine infusion in sham groups had no cardiac protection effect (78.2 ± 2.3%, *n* = 5) (Fig. 2c). Thus, maintenance of chronic pain status is not required for the SNI-induced cardioprotection.

### Inhibition of ERK activation in PVA reduces cardioprotection

**Fig. 3:**
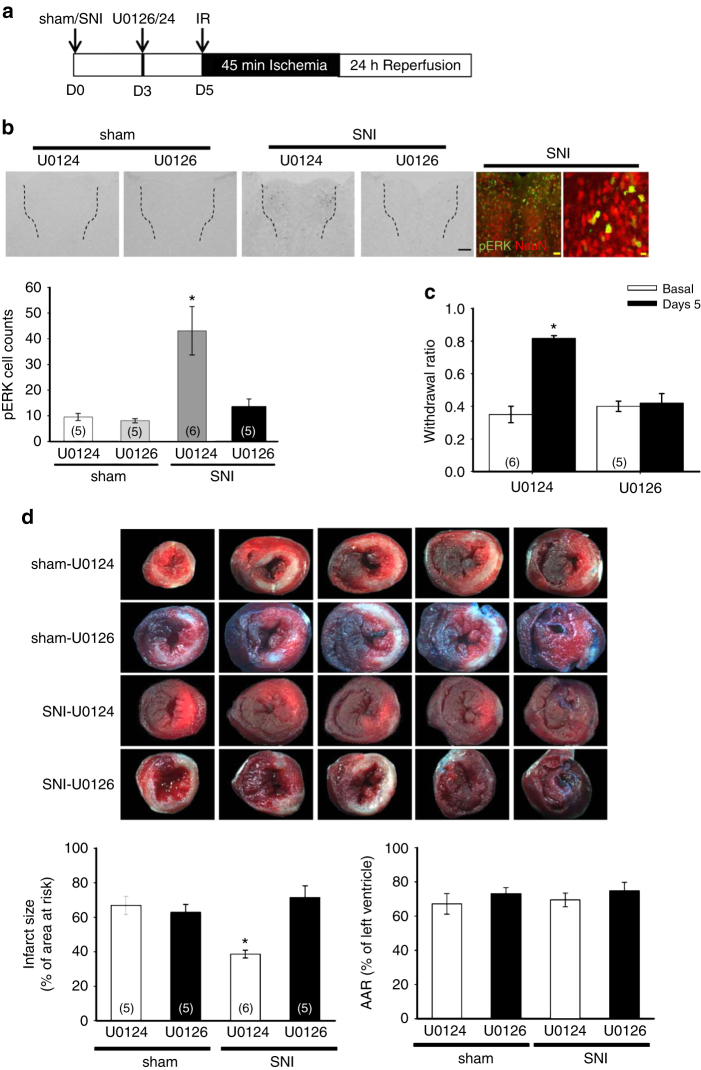
Infusion of U0126 but not U0124 in PVA blocks the SNI-induced cardioprotection. **a** Schematic of experimental design showing the timeline for sham/SNI surgery (D0), intra-PVA infusion of U0126/or U0124 (1.5 mM in 0.3 μL 50% DMSO, D3) and myocardial IR surgery (D5). **b** Immunohistochemical staining of pERK, an indication of ERK activation, in PVA (outlined by *dashed line*) from sham and SNI animals received U0126 or U0124 infusion. *Scale bar* = 100 μm. Immunofluorescent imaging of pERK (*green*) and NeuN (*red*) in PVA from SNI animal. *Scale bar* = 50 μm (lower magnification) and 10 μm (higher magnification), respectively. Quantification results of pERK-positive cells in PVA from these animals. **c** Mechanical responses of hind paws at D0 (basal) and D5, after intra-PVA infusion of U0124 or U0126 (D3). *p < 0.05 vs. basal group. **d** Representative images of TTC staining in heart cross-sections. Quantification results of infarct size and AAR in cardiac sections from U0124 and U0126-infused animals (*middle* and *lower panels*). Infusion of U0126 prevented the SNI-induced cardioprotection. **p < 0.001 vs. U0124 group. *Error bars* indicate SEM. Statistical significance was determined by one-way ANOVA (**b**, **d**) or two-way RM ANOVA (**c**)

Intrathecal infusion of lidocaine did not reduce SNI-dependent cardioprotection also suggests that this cardioprotective signal is not originated from the spinal cord level. Previous studies showed changes in higher brain centers are important for maintaining and/or developing chronic pain^31–33^. Thus, we hypothesized the SNI-induced cardioprotective signal is originated from brain regions involved in central sensitization in chronic pain. Mitogen-activated protein kinase phosphorylates and activates ERK and plays a critical role in organizing neural plasticity^34^. ERK activity in the spinal cord plays an important role in transmitting the nociceptive signal and in central sensitization^35^. From our previous study, ERK activity in the anterior part of the paraventricular thalamus (PVA) is required for maintaining chronic muscular pain^36^. We have recently shown that inhibition of ERK activity in PVA attenuated SNI-induced neuropathic hyperalgesia^37^. To test whether PVA is involved in the SNI-induced cardioprotection, we infused U0126 (1.5 nmol), a MEK inhibitor, or its inactive analog, U0124 as a negative control for U0126, into PVA. U0126 or U0124 was infused 3 days after SNI or sham operation and mice were subjected to IR injury 2 days after PVA infusion (Fig. 3a). This concentration of U0126 reduces formalin-induced and acid-induced mechanical hyperalgesia when applied in amygdala or PVA, respectively^36, 38^. U0126 but not U0124 infusion decreased the number of pERK-positive cells in PVA (Fig. 3b) and attenuated mechanical hyperalgesia induced by SNI at day 5 (Fig. 3c). PVA infusion of U0126 or U0124 in sham groups did not induce any observable pERK-positive cells in PVA (Fig. 3b). Most of the pERK signals were co-localized with NeuN-positive signals, which indicates PVA neurons were activated in the SNI-induced neuropathic pain mice (Fig. 3b). We then examined the effect of U0126 on SNI-induced cardioprotection. The results demonstrated that intra-PVA infusion of U0126 but not U0124 abolished SNI-induced cardioprotection (Fig. 3d). The infarct size of the U0124 group (38.6 ± 2.4%, *n* = 6) was similar to the SNI group shown in Fig. 1b. In contrast, the infarct size of U0126 group (71.4 ± 6.8%, *n* = 5) was comparable to those of naïve or sham group (Fig. 3d). Serum CKMB levels were also significant higher in U0126 compared to U0124 groups (Supplementary Fig. 3). The infarct sizes of U0124 and U0126 infusion in sham groups were similar to the sham group shown in Fig. 1b. There was no significant difference in the histology and fibrosis between U0124 and U0126 infusion in sham groups (Supplementary Fig. 4A). Thus, inhibition of ERK activity in PVA abolishes the SNI-induced cardioprotection.

### Increased PVA neuronal activity in SNI animals

**Fig. 4:**
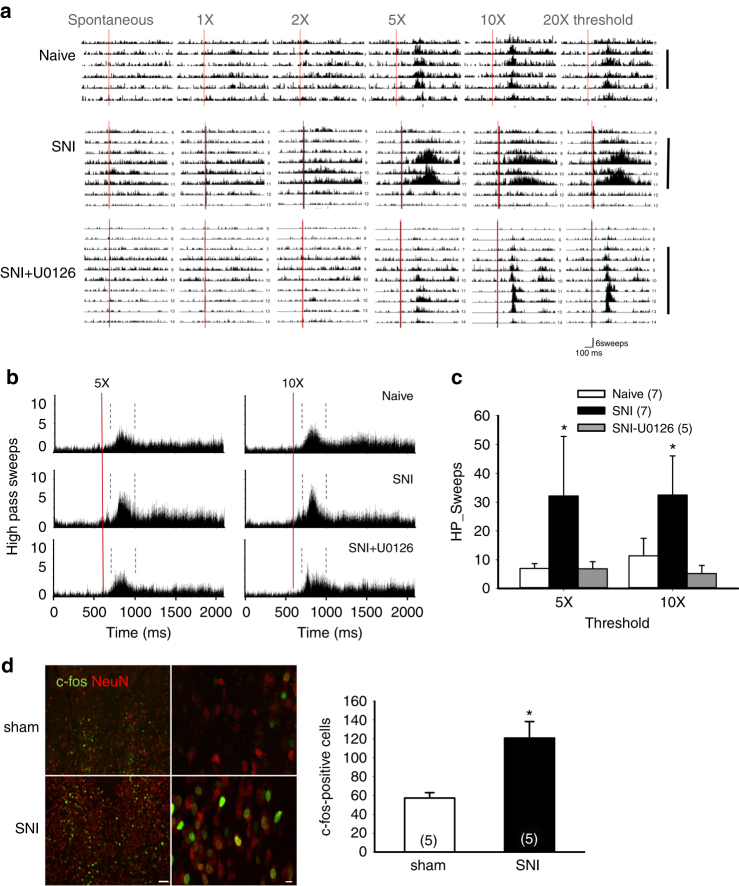
Neuronal activities of PVA increase in SNI-induced neuropathic pain model. **a** Representative sweeps of spike firing of PVA neurons in naïve mouse, SNI, and SNI with intra-PVA U0126 infusion groups in response to different strength of stimulations. The *red line* indicates the electrical stimulation on the left sciatic nerve. There is no current input in the spontaneous recording. The *black line* on the right indicates those channels in PVA region. **b** The average of evoked sweep spike summation from 5 to 7 mice in each experimental groups responding to the stimulation current 5 and 10 times to the threshold, respectively. PVA neuron sweep spike are segregated into FC, and LC defined by the spike pattern (responding to the stimulation faster or slower than 400 ms). The first and second *dash lines* indicate 100 and 400 ms after electrical stimulation, respectively. **c** Sweep spikes of 0–100 ms FCs in different groups. The summation of sweep spikes responding to different strength of electrical stimulus of each component was analyzed by one-way ANOVA followed a post hoc testing method. *p < 0.05 compared to naïve group and to SNI-U0126 group. **d** Immunofluorescent imaging of c-fos (*green*) and NeuN (*red*) in PVA from sham and SNI animals. *Scale bar* = 50 μm (*left panel*) and 10 μm (*right panel*), respectively. *p < 0.05 compared to sham group. *Error bars* indicate SEM. Statistical significance was determined by Student’s *t-*test (**d**)

To examine whether SNI led to electrical remodeling of PVA, we recorded PVA neuronal activity using a multichannel probe in anesthetized mice with electric current stimulations on left sciatic nerve. The exact channels inserted into PVA were identified via the post hoc histological examination of lesions marks. In vivo recording revealed that PVA neurons were excited by noxious stimuli in a strength-dependent manner in naïve mice, indicating PVA is indeed involved in the nociception circuitry (Fig. 4a). The evoked sweep spike unit of PVA neuron in naïve mice (Fig. 4b) patterned with two components: the fast-responding component (FC), responded to sciatic nerve stimulus started from 100 to 400 ms, and the late component (LC), responded to stimulus later than 400 ms. The peak responding time for the FC was 200–300 ms after stimulus. FC showed a trend of increase of sweep numbers in SNI group compared to naïve group (Fig. 4c). Interestingly, there was a new response component (100 ms-FC) recorded within 100 ms after sciatic stimulation in SNI-induced chronic hyperalgesia mice (Fig. 4b, c). The increase of spike units and the new 100 ms-FC indicate a switch of firing pattern under noxious stimulus in SNI-group, which suggests a neuronal plasticity switch at the chronic phase after nociception induction. We also examined the expression of c-fos as a marker for neuronal activation. A significant increase in the c-fos-positive neurons in PVA was observed in SNI compared to sham group 4 h after surgery (Fig. 4d). Together, these results demonstrate that PVA neurons are activated by stimulation on the peripheral nerve and their activities are further enhanced in SNI model.

### Direct activation of PVA neurons provides cardioprotection

**Fig. 5:**
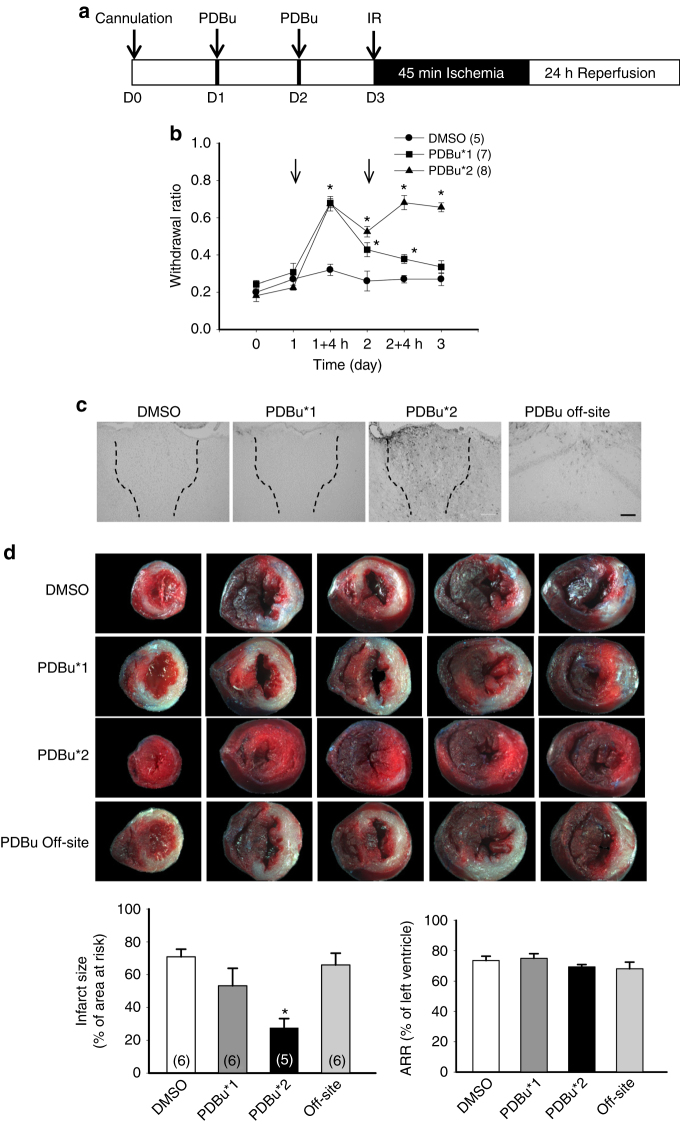
Infusion of PDBu in PVA induce cardioprotection in naïve animals. **a** Schematic of experimental design showing the timeline for cannulation (D0), intra-PVA infusion of PDBu (20 pmol in 0.3 μL 50% dimethyl sulfoxide (DMSO)) or 50% DMSO (D1, D2), and myocardial IR surgery (D3). **b** Mechanical responses of hind paws at D0 (basal), D1, D1 4 h after first PDBu infusion, D2, D2 4 h after 2nd PDBu infusion and D3. *p < 0.05 vs. DMSO group. *Arrow* indicates intra-PVA infusion of PDBu. **c** Immunohistochemical staining of pERK in PVA (outlined by *dashed line*) from animals received PDBu and DMSO infusion. *Scale bar* = 100 μm. PDBu*1, infusion of PDBu (D1) and infusion of 50% DMSO (D2) in PVA. PDBu*2, infusion of PDBu at D1 and D2 in PVA. PDBu off-site, infusion of PDBu at D1 and D2 in CA1 region of hippocampus. *Scale bar* = 100 μm. **d** Representative images of TTC staining in heart cross-sections. Quantification results of infarct size and AAR in cardiac sections from different treatment groups (*lower panel*). Two infusions of PDBu in PVA were sufficient to induce cardioprotection in naïve animals. *p < 0.05 vs. DMSO group. *Error bars* indicate SEM. Statistical significance was determined by two-way RM ANOVA (**b**) or one-way ANOVA (**d**)

We next asked whether pharmacological activation of ERK in PVA also has an effect on cardioprotection without peripheral neuronal injury. We infused PDBu (20 pmol), a protein kinase C activator, into PVA either once or twice before IR injury (Fig. 5a). Infusion of PDBu once induced to a transient hyperalgesia; interestingly, infusion of PDBu twice induced a sustained hyperalgesia (Fig. 5b). Activation of ERK in PVA was examined by immunostaining after IR injury and the results showed that repeated PDBu infusion increased the pERK-positive signals compared to DMSO or single PDBu infusion groups (Fig. 5c). Mice received repeated PDBu infusion exhibited a significant smaller infarct size (27.6 ± 5.7%, *n* = 5) compared to DMSO group (70.9 ± 4.7%, *n* = 6). These results demonstrated that infusion of PDBu into PVA, presumably via activation of ERK, induces cardioprotection in the absence of peripheral neuronal injury.

### Stimulation in PVA by optogenetic elicits cardioprotection

**Fig. 6:**
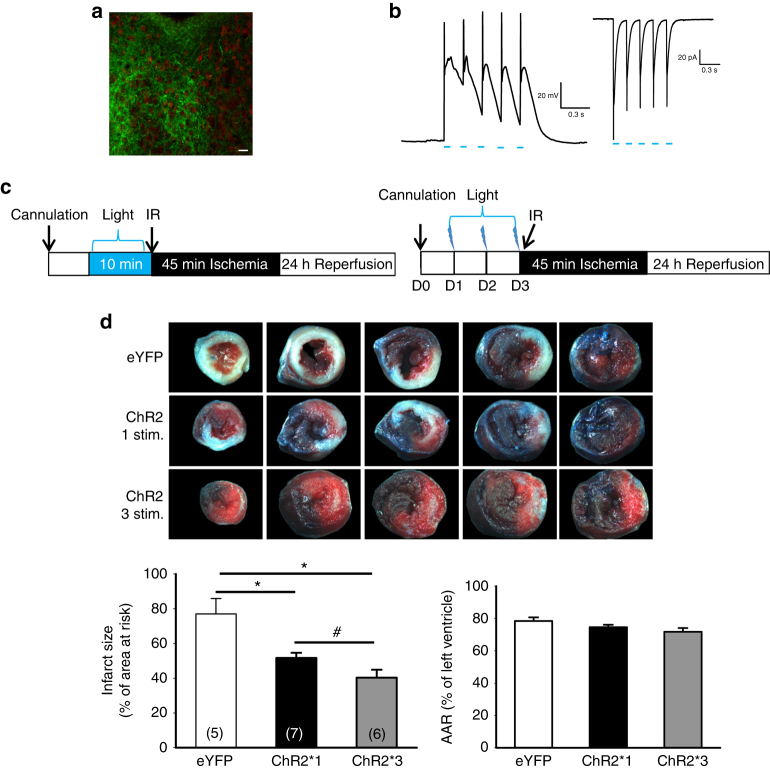
Optogenetic activation of PVA neurons induces cardioprotection. **a** Immunofluorescent images of hChR2(H134R)-EYFP-positive signals in PVA regions, brain section was co-stained with neuronal marker, NeuN (red signal) (*scale bar* = 20 μm). **b** Representative action potential recording of hChR2(H134R)-EYFP-positive neurons in PVA elicited by blue light stimulation (5 mW mm^−2^, 5 Hz, 10 ms pulse indicated by *blue lines*) (*left panel*). Inward currents elicited by blue light stimulation from hChR2(H134R)-EYFP-positive neurons (*right panel*). **c** Schematic of experimental design showing the timeline for single and repeated blue light stimulation and myocardial IR surgery. **d** Representative images of TTC staining in heart cross-sections. Quantification results of infarct size and AAR in cardiac sections from EYFP and ChR2 one stimulation (ChR2 1 stim.) and ChR2 three stimulations (ChR2 3 stim.) groups. Optogenetic stimulation of neurons in PVA for 10 min or 3 consecutive days prior to IR surgery protected heart from IR injury. *p < 0.05 vs. eYFP group. #*p* = 0.05. *Error bars* indicate SEM. Statistical significance was determined by one-way ANOVA (**d**)

To further determine the role of PVA in cardioprotection, we activated neurons using optogenetic tool by expressing channelrhodopsin 2 (ChR2) in PVA. The expression of ChR2 was detected by examining the fluorescent signals in PVA neurons after 6–8 weeks infection of AAV-CaMKIIα-hChR2 (H134R)-eYFP vector (Fig. 6a). To test whether the expressed ChR2 is functional, we performed single cell recording from PVA brain slice. As shown in Fig. 6b, blue light stimulation induced inward currents and evoked action potential in PVA neurons. We next investigated whether optogenetic activation of PVA neurons could induce cardioprotection. One group of mice was subjected to blue light stimulation in the PVA 10 min prior to IR injury procedure. Another group was subjected to blue light stimulation in the PVA for 10 min in 3 consecutive days before IR injury (Fig. 6c). Using this approach, we demonstrated that both single and repeated optogenetic stimulations reduced the infarct size of ChR2-expressing group (51.7 ± 2.9%, *n* = 7 and 40.3 ± 4.6%, *n* = 6, respectively) compared to eYFP-expressing group (77.0 ± 8.9%, *n* = 5) (Fig. 5d). Repeated optogenetic stimulations further reduced the infarct size compared to that of single optogenetic stimulation (*p* = 0.05). Histological examination of the cardiac transverse section 4 weeks after IR injury also showed reduced fibrosis and preserved left ventricular free wall in repeated optogenetic stimulation Ch2R group compared to eYFP groups (Supplementary Fig. 4B). Repeated optogenetic stimulations also lead to ERK activation in PVA (Supplementary Fig. 5). Thus, direct activation of PVA neurons via optogenetic stimulation induces cardioprotection.

### Inhibiting PVA neurons prevents SNI-induced cardioprotection

**Fig. 7:**
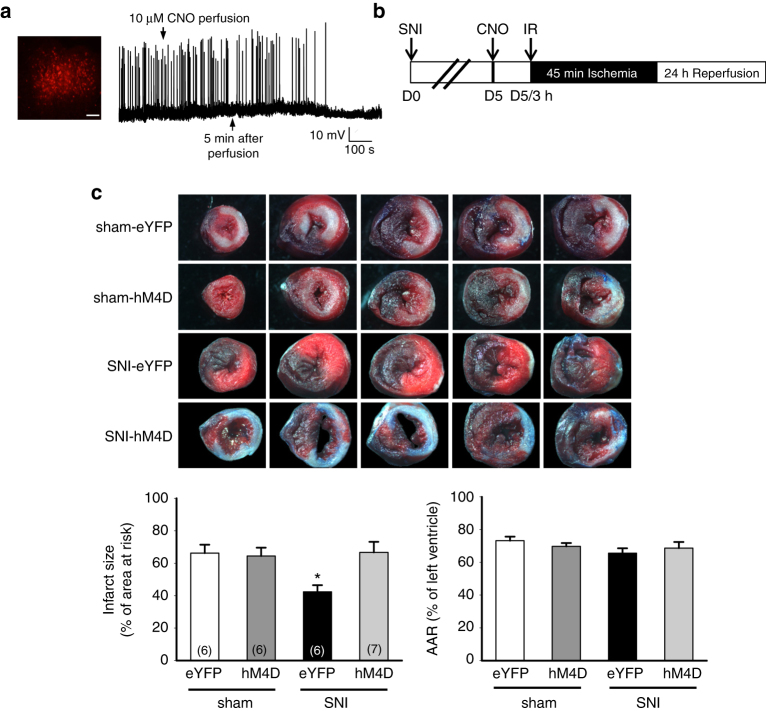
Inhibition of PVA neuron with DREADD alleviates SNI-induced cardioprotection. **a** Immunofluorescent images of AAV-hM4D-mCherry (*red*)-positive signals in PVA regions (*scale bar* = 100 μm). Evoked spontaneous action potentials recorded from hM4D-mCherry-positive PVA neurons with CNO bath perfusion. **b** Schematic of experimental design showing the timeline for SNI (D0), CNO IP injection (D5) and IR injury (3 h after CNO injection). **c** Representative images of TTC staining in heart cross-sections. Quantification results of infarct size and AAR in cardiac sections from eYFP and hM4D groups in sham and SNI animals. Activation of hM4D with CNO alleviates SNI-induced cardioprotection. *p < 0.05 vs. eYFP group. *Error bars* indicate SEM. Statistical significance was determined by one-way ANOVA (**c**)

To ensure that PVA is directly involved in SNI-induced cardioprotection, we applied designer receptors exclusively activated by designer drugs (DREADD) technique to inhibit PVA neuronal activity remotely^39^. We expressed Gi-coupled DREADD (AAV-CaMKII-hM4D-mCherry) or control eYFP in PVA. We confirmed the expression of hM4D by the fluorescent mCherry signal in PVA 4 weeks after injection (Fig. 7a). To ensure the expressed hM4D did inhibit PVA neuronal activity, we recorded hM4D-mCherry-positive neurons in brain slices in the presence of hM4D agonist, clozapine-N-oxide (CNO). Our recording showed that the evoked spontaneous action potential slowed down and eventually stopped after CNO perfusion in hM4D-mCherry-positive PVA neurons (Fig. 7a). We next examined whether DREADD-mediated inhibition of PVA could attenuate SNI-induced cardioprotection. The experimental procedure was outlined in Fig. 7b. Intraperitoneal (IP) injection of CNO (3 mg/kg) abolished SNI-induced cardioprotection (Fig. 7c). The infarct size of the eYFP group (42.4 ± 4.15%, *n* = 6) was similar to the SNI group shown in Fig. 1b. In contrast, the infarct size of hM4D group (66.6 ± 6.6%, *n* = 7) was comparable to those of naïve or sham group (Fig. 7c). The infarct sizes were similar in sham animals expressing eYFP or hM4D in PVA (Fig. 7c). Thus, silencing PVA neuronal activity with Gi-coupled DREADD efficiently attenuates SNI-induced cardioprotection.

### Ganglionic blockers inhibit SNI-induced cardioprotection

**Fig. 8:**
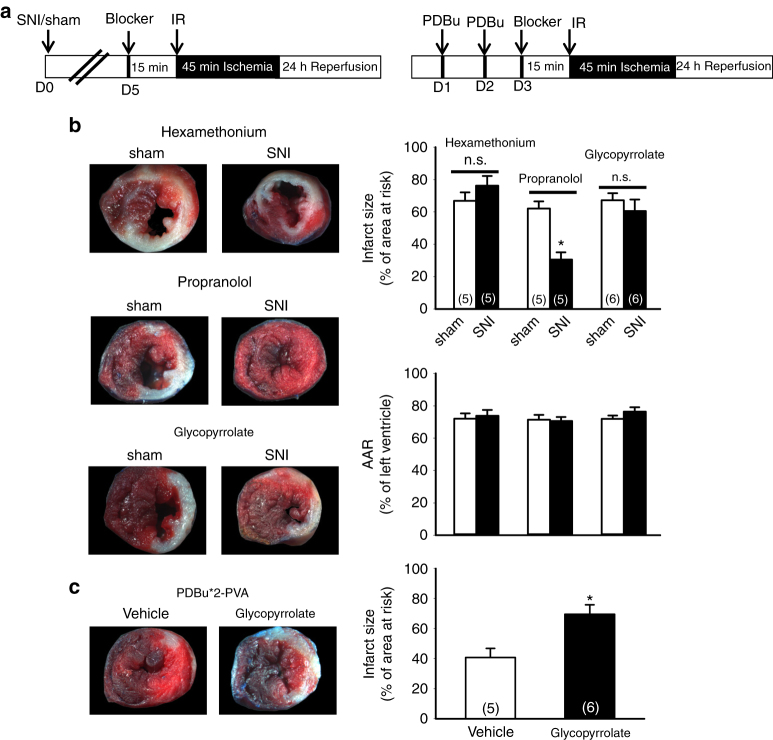
Parasympathetic but not sympathetic blockers prevent SNI-induced and PVA PDBu infusion-induced cardioprotection. **a** Schematic of experimental design showing the timeline for SNI surgery (D0), i.v. infusion of blockers (D5), and myocardial IR surgery (D5) (*left panel*). *Right panel* shows the timeline for PVA PDBu infusion (D1, D2), i.v. infusion of blockers (D3), and myocardial IR surgery (D3). **b** Representative images of TTC staining in heart cross-sections from different treatment groups (*left panels*). Quantification results of infarct size and AAR in cardiac sections different groups (*right panels*). Hexamethonium (30 mg/kg), propranolol (2 mg/kg), glycopyrrolate (0.4 mg/kg) were infused i.v. 15 min prior to myocardial ischemia surgery in mice received sham or SNI surgery. *p < 0.05 vs. sham group. Hexamethonium and glycopyrrolate but not propranolol prevented SNI-induced cardioprotection. **c** Representative images of TTC staining in heart cross-sections in mice treated with vehicle or glycopyrrolate (*left panels*). Quantification results of infarct size in cardiac sections (*right panels*). *p < 0.05 vs. vehicle group. *Error bars* indicate SEM. Statistical significance was determined by Student’s *t-*test (**b**, **c**)

Sympathetic and parasympathetic nervous systems are known to involve in cardioprotection provided by remote preconditioning^7, 26^. To test whether autonomic nerves are involved in the SNI-induced cardioprotection, we injected hexamethonium (30 mg/kg), an autonomic ganglionic blocker, before IR surgery (Fig. 8a). Treatment of hexamethonium reversed the SNI-induced cardioprotection effect. There is no significant difference between the infarct size of SNI (76.2 ± 6.0%, *n* = 5) and sham (66.9 ± 5.3%, *n* = 5) groups after treatment of hexamethonium, which suggests the involvement of autonomic nervous system in SNI-induced cardioprotection. We then used propranolol (2 mg/kg) and glycopyrrolate (0.4 mg/kg) to inhibit sympathetic and parasympathetic nervous systems, respectively. Treatment of propranolol has no effect on the SNI-induced cardioprotection, the infarct size was 62.1 ± 4.5% (*n* = 5) vs. 30.6 ± 4.5% (*n* = 5) in sham and SNI groups, respectively. In contrast, glycopyrrolate abolished SNI-induced cardioprotection. There was no significant difference between the infarct size of SNI (67.2 ± 4.3%, *n* = 6) and sham (60.5 ± 7.2%, *n* = 6) groups after treatment of glycopyrrolate (Fig. 8b). Serum CKMB levels were significantly higher in glycopyrrolate compared to propranolol groups (Supplementary Fig. 3). We also investigated whether the cardioprotection of PDBu infusion is also dependent on parasympathetic nervous system. Mice received two PDBu infusions in PVA were subjected to myocardial IR injury and glycopyrrolate was injected 15 min prior to IR surgery (Fig. 8a). Treatment of glycopyrrolate also attenuated cardioprotection induced by PDBu infusion, the infarct sizes was 40.7 ± 6.2% (*n* = 5) vs. 69.5 ± 6.3% (*n* = 6) in the vehicle and glycopyrrolate groups, respectively. The whole series of TTC stained cardiac sections were shown in Supplementary Fig. 6. These results demonstrated that parasympathetic but not sympathetic nerve system is involved in SNI-induced cardioprotection. Parasympathetic nervous system also mediates the cardioprotection induced by direct activation of PVA neurons in the absence of peripheral tissue damage.

### SNI and stimulation of PVA lower heart rate

**Fig. 9:**
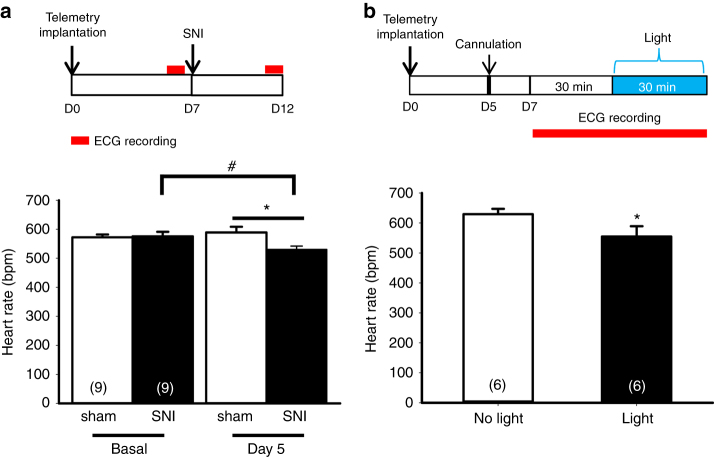
SNI surgery and optogenetic activation of PVA neurons reduce heart rates in mice. **a** Schematic of experimental design showing the timeline for telemetry probe implantation (D0), ECG recording before SNI surgery (basal at D7), and ECG recording 5 day after SNI surgery (D12) (*upper panel*). Heart rates of animals subjected to sham or SNI surgery at basal or 5 days after surgery (*lower panel*). SNI surgery lead to a reduced heart rate compared to sham group or its own basal level. *p < 0.05 vs. sham group at day 5, #*p* < 0.05 vs. SNI basal state. **b** Schematic of experimental design showing the timeline for telemetry probe implantation (D0), cannulation (D5), and ECG recording for 30 min before and during blue light stimulation (D7) (*upper panel*). These animals were infused with AAV-CaMKIIα-hChR2 (H134R)-eYFP vector in PVA for 6–8 weeks before telemetry implantation. Optogenetic stimulation of PVA neurons lead to a reduction in heart rate compared to that of the same animal without light stimulation (*lower panel*). *p < 0.05 vs. no light stimulation. *Error bars* indicate SEM. Statistical significance was determined between sham and SNI group by Student’s *t-*test (**a**) or intragroup by Paired *t-*test (**a**, **b**)

**Fig. 10:**
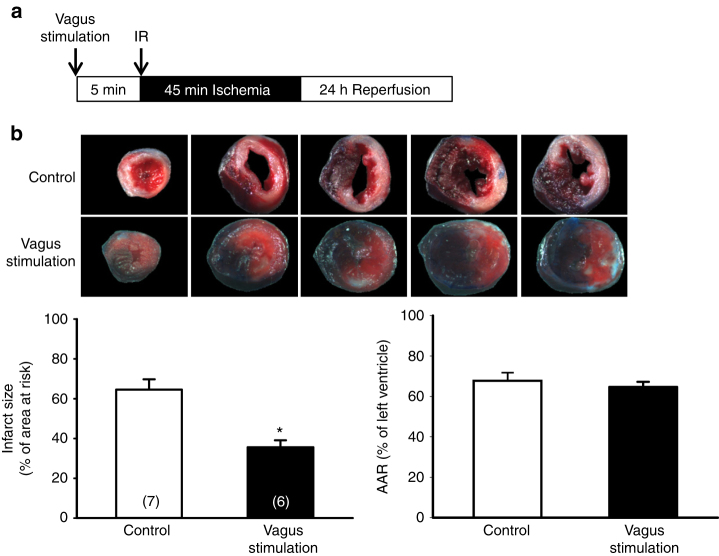
Direct stimulation of vagus nerve protects heart from IR injury. **a** Schematic of experimental design showing the timeline for vagus nerve stimulation (5 min) and IR injury (immediately after vagus nerve stimulation). **b** Representative images of TTC staining in heart cross-sections. Quantification results of infarct size and AAR in cardiac sections from vagus stimulation and control groups. Activation of vagus nerve provides cardioprotection. *p < 0.05 vs. control group. *Error bars* indicate SEM. Statistical significance was determined by Student’s *t-*test (**b**)

The main parasympathetic nerve that innervates to heart is vagus nerve. We reasoned that if activation of the vagus nerve is involved in the cardioprotection, we should be able to detect decrease of heart rate. To test this, we measured the heart rate using radiotelemetry probes in conscious mice subjected to SNI or optogenetic stimulation of PVA. The heart rate was measured at basal level before and 5 days after sham or SNI surgery. At the basal level, there was no difference in the heart rate between the two groups. Interestingly, we detected a significant decrease in the heart rate 5 days after SNI surgery (530 ± 12 bpm, *n* = 9) compared to their basal level (576 ± 15 bpm, *n* = 9) (Fig. 9a). There was also a significant difference between the SNI group and sham group (589 ± 19 bpm, *n* = 9) 5 days after surgery. To test whether direct optogenetic activation of PVA could reduce heart rate, we measured the heart rate before and after optogenetic stimulation of PVA neurons. The results showed that optogenetic stimulation reduced the heart rate (629 ± 18 vs. 555 ± 33 bpm before and after stimulation, *n* = 6) (Fig. 9b). To provide direct evidence that activation of vagus nerve induces cardioprotection in our model, we electrically stimulated the left vagus nerve for 5 min prior IR injury (Fig. 10a). The results demonstrated that direct vagus stimulation induced cardioprotection (Fig. 10b). The infarct size of the control group (64.6 ± 5.2%, *n* = 7) was comparable to those of naïve or sham group. In contrast, the infarct size of vagus stimulation group (35.6 ± 3.5%, *n* = 6) was similar to the SNI group shown in Fig. 1b. Together, these results suggest that SNI and optogenetic stimulation in PVA induce cardioprotection via vagus nerve.

## Discussion

In this study, we demonstrated that chronic neuropathic pain induced cardioprotective effect against IR injury in mice. It has been shown an abdominal incision either 15 min or 24 h before coronary occlusion reduces the infarct size^40^. This cardioprotection initiated by skin nociception, requires intact spinal cord and activation of cardiac sensory and sympathetic nerves. Topical application of capsaicin, which activates C sensory fibers, also induces cardioprotective effect^7^. Little is known concerning the effect of chronic pain on cardioprotection. Clinically, prodromal angina is associated with increased cardioprotection in patients who received percutaneous coronary intervention during ST-elevation myocardial infarction^13, 14^. The angina-associated cardioprotection is thought to represent a clinical correlation of IPC. Based on the current finding, it is possible that angina may induce cardioprotection via a nociceptive signal pathway.

Our study showed that SNI neuropathic pain induces cardioprotection against IR injury in mice. However, several studies show a positive correlation between chronic pain and CVD risk in human^20–24^. There could be several reasons for the discrepancy. We investigated the effect of SNI-induced neuropathic pain on cardioprotection against IR injury. Most of these studies include a wide spectrum of CVD risk, range from hypertension, coronary heart disease, pulmonary heart disease and diseases of pulmonary circulation, other forms of heart disease, cerebrovascular disease, and diseases of arteries to diseases of veins. Moreover, chronic pain and CVD was ascertained by self-report and also the severity and types of chronic pain are different. Potential confounding factors such as physical activities, metabolic diseases, and vitamin D deficiency in chronic musculoskeletal pain patients could be associated with CVD risk. In these retrospective case-control studies, it is difficult to confirm the causality between chronic pain and CVD risk. There is one cohort study showing self-reported chronic widespread pain and chronic regional pain are associated with hospitalization due to ischemic heart disease, cerebrovascular disease, and infection^41^. It will be important for prospective cohort studies to further investigate the causality between chronic pain and prognosis of MI patients receiving reperfusion therapy.

Most preconditioning cardioprotection studies were conducted minutes or hours before IR injury^42^. Some studies investigated the effect of a delay or chronic preconditioning on cardioprotection. For example, angiotensin II triggers early (within 1 h) and delayed pharmacological preconditioning (after 24 h and up to 72 h, but not 96 h) in isolated Langendorff perfused hearts^43^. Daily limb IPC for 15 min, 24 h, or 9 days in mice provides similar cardioprotection in a global IR injury model using isolated hearts^15^. This study suggests that cardioprotection could be achieved by chronic IPC. Also, a non-IPC resulted from abdominal incision 1 day before IR injury provides cardioprotection^40^. Peripheral nociception induced by skin incisions on the abdomen also provided cardioprotection in rodent^7^. This study demonstrated acute nociceptive stimulation induces early remote preconditioning against myocardial infarction. However, whether chronic non-IPC could provide cardioprotection is unclear. In current study, IR injury was conducted 5 days after SNI surgery when the chronic mechanical hypersensitivity was developed. Our time point certainly fit into the delayed/chronic remote preconditioning on cardioprotection. To our knowledge this is the first study to demonstrate that chronic remote nonischemic stimulation provides cardioprotection against IR injury.

By intrathecal infusion of lidocaine, we showed that maintenance of chronic pain status and interrupting the nociceptive transduction through spinal cord are not required for the SNI-induced cardioprotection. These results also suggested that supraspinal regions are involved in regulating this cardioprotection. Many brain regions are involved in the perception of pain, including parabrachial, amygdala, thalamus, cortex, nucleus accumbens (NAc), anterior singular cortex (ACC), periaqueductal gray, and rostral ventromedial medulla (RVM)^44, 45^. Anterior nucleus of the paraventricular thalamus (PVA) is located in the midline thalamus. PVA plays a role in regulating negative emotion behavior and stress^46^. It connects to pain-related brain regions such as amygdala, NAc, and ACC^46^. We have previously shown that ERK activity in PVA is required for acid-induced chronic muscle pain^36^ and other chronic pain^37^. Here, we validated the role of PVA in chronic neuropathic pain-induced cardioprotection by providing the following evidences. First, inhibition of ERK activity in PVA by U0126 infusion abolished the SNI-induced cardioprotection. Second, direct activation of PVA neurons by PDBu or optogenetic stimulation is sufficient to induce cardioprotection in the absence of peripheral tissue damage. Infusion of PDBu into other brain region, such as hippocampus did not induce cardioprotection, which suggests the specific role of PVA in cardioprotection. Third, inhibition of PVA neuronal activity using DREADD approach abolished the SNI-induced cardioprotection.

Humoral factors and neurogenic pathway are involved in the relay pathways of RIPC-induced cardioprotection^27, 47, 48^. Cardioprotection induced by brief IR of the anterior mesenteric artery was abolished by hexamethonium, a ganglion blocker that provides evidence that neural pathway mediates this cardioprotection^5^. Blockage of parasympathetic nerve by vagotomy or atropine abolishes RIPC-induced cardioprotection, which indicates the importance of parasympathetic nervous system^26, 49^. Our results showed that SNI-induced cardioprotection was prevented by hexamethonium and glycopyrrolate but not propranolol, which indicates parasympathetic nerve system mediates the SNI-induced cardioprotection. Direct activation of PVA neurons by PDBu or optogenetic stimulation also induced cardioprotection in the absence of peripheral tissue damage and this protection was also prevented by glycopyrrolate. Release of humoral factors of RIPC is dependent on prior activation of vagus nerve^47^. Humoral factors of RIPC are produced by the visceral organs innervated by the posterior gastric branch of the vagus nerve^48^. Although we cannot exclude the involvement of humoral factors in the SNI-induced cardioprotection, it is possible that activation of vagus nerve triggers the release of humoral factors in the PVA-dependent cardioprotection in IR injury.

The exact connection between PVA and parasympathetic nerve system is unclear. Previous tracing studies demonstrate that PVA sends projections to many brain regions, including amygdala and the bed nucleus of the stria terminalis (BNST) in rats and monkeys^50–52^. BNST projects to the nucleus tractus solitarii (NTS) and nucleus ambiguus and modulates baroreflex response via parasympathetic nerve system^53–55^. Direct activation of the brainstem dorsal motor nucleus of the vagus nerve (DVMN) induces cardioprotection against IR injury^26^. It is possible that activation of PVA neurons affect DVMN via the BNST/NTS/nucleus ambiguus connection. Another pain-related brain region, RVM also connects to NTS. We could not rule out the possibility that PVA influences NTS activity via RVM or other brain regions.

The role of the parasympathetic nervous system in cardioprotection against IR injury has been studied in several species. For example, infarct size reduction in an ex vivo perfused rat heart by vagal stimulation^25^, direct pre-ganglionic parasympathetic neuron activation induced cardioprotection in rat^26^, and abolishment of RIPC and cardioprotection due to vagotomy or muscarinic cholinergic blockade in rabbit^56^. Vagus nerve stimulation provides significant cardioprotection even applied 30 min after LA occlusion in swine^57^. Similarly, we showed that direct vagus nerve stimulation can also provide cardioprotection against IR injury in murine (Fig. 10). It has been shown that vagal stimulation and acute ischemia increase the myocardial interstitial acetylcholine (ACh) level and protect heart from IR injury^58^. ACh protects IR injury via activation of muscarinic receptors and modulation of mitochondrial K (ATP) channels^59–62^. We demonstrated that the myocardium infarct size, TUNEL-positive cells, and the level of cleaved (activated) caspase 3 were all reduced in mice subjected to SNI. It has been shown that translocation of PKCε from cytosol to membrane fraction, an indication of PKCε activation, is essential for RIPC by activating mitochondria potassium ATP channel and this activation can be abolished by hexamethonium^29, 63^. We also demonstrated that SNI but not sham surgery increased membrane fraction of PKCε in the mouse heart. Taken together, our results support the idea that chronic neuropathic pain activates PVA neurons which then increase the vagus nerve activity, release of ACh and activation of PKCε, and thus protect the cardiac myocytes from IR injury.

## Methods

### Animal preparation

All animal procedures conformed to National Institutes of Health guidelines in accordance with the guidelines specified by the Institutional Animal Care and Utilization Committee, Academia Sinica (Taipei, Taiwan). Wild-type C57BL/6JNarl mice were obtained from National Laboratory Animal Center. Male mice (22–27 g; 8–12 weeks) were used for all studies. Mice were under 12:12-h light-dark cycles with food and water available ad libitum. Mice were assigned to experimental groups randomly. The investigator was blind to different drugs treatment before cardiac surgery.

### Chronic neuropathic pain model

SNI was conducted as previously described^64^. In brief, mice were anesthetized by vaporized isoflurane (1.5%) and the three distal branches of the sciatic nerve were exposed carefully without damaging the muscle bundles. The common peroneal and sural nerves were tightly ligated by 8-0 nylon sutures. Approximately a 2-mm length segment of each ligated nerve was dissected distally to the ligature without stretching or touching the tibia nerve. In the sham group, the suture went underneath the nerve without ligating or dissecting the nerve. Five days after SNI or sham surgery, mice were subjected to IR injury. If there is no enhanced mechanical hyperalgesia in the 2nd day after surgery, this animal was excluded.

### Von Frey filament behavioral assay

Mice were placed in transparent plastic cubicles on a wire mesh-bottomed platform and habituated for 15–20 min before testing. To measure the withdrawal response to mechanical stimuli of the test mice, 1 g of bending force of von Frey monofilament was applied until the hindpaws were withdrawn. The percentage response for each foot was obtained by the number of withdrawal responses of 10 applications to each foot.

### Myocardial IR injury

Mice were anesthetized by IP injection of ketamine (87.5 mg/kg) and xylazine (12.5 mg/kg). After intratracheal intubation, the respiration of the animal was controlled by a small animal ventilator (tidal volume 0.22 ml and a respiration rate 90/min) with a continuous supply of 0.25–0.5% isoflurane. Left-sided thoracotomy was performed by incising the muscle at the 3rd intercostal space. The LAD artery was ligated together with a silicon tube by 7-0 silk suture. A successful LAD ligation will lead to a paleness appearance in the ischemia myocardium. Saline moisture cotton was placed on the surface during the ischemia period (45 min) to avoid tissue drying. After 45 min, the silicon tube was removed to allow reperfusion and the suture was left uncut. The chest was closed in layers and air was drained out of the thorax using 1 ml syringe. Mice were put back in the recovery cage until they wake up.

Infarct size was determined 24 h after reperfusion. Mice were deeply anesthesia and perfused with PBS. LAD was re-ligated using the uncut suture and 1% Evans blue dye was infused into the heart via the aorta to distinguish the myocardium at risk (the AAR) and non-ischemic area. The heart was isolated and frozen for 20 min at −20 °C and then transversally sectioned at 2 mm thick and each slice was weighed. We normally obtained four sections from each heart, the upper section containing aorta root was not influenced by ischemic damage and was not processed. The lower three sections were incubated with 1% 2,3,5-TTC for 10 min at 37 °C. The entire series of TTC stained cardiac sections were shown in the figures. The first row of the images were apex section, the other four rows were images from both sides of the other two cardiac sections. Pale region was regarded as area of necrosis (AON). AON and AAR were calculated as the average percent area per slice from both sides of each section. Then they were normalized to slice weight respectively by following this equation: weight of total AAR = (Weight of slice 1 × % AAR of slice 1) + (Weight of slice 2 × % AAR of slice 2) + (Weight of slice 3 × % AAR of slice 3). AON weight was calculated in the same manner. Finally, infarcted size was expressed as a percentage of the weight of AON to the weight of AAR and the AAR was the ratio of the left ventricle^65^. If the AAR is >90%, this animal was excluded.

### Protein extraction and immunoblotting

Left ventricles were dissected out from mice 5 days after SNI surgery and homogenized in buffer A (in mM: 5 Tris, 4 EGTA, 2 EDTA, 5 DTT, 1 PMSF) and containing EDTA-free protease inhibitor and vortex every 5 min and stood on ice for 20 min. The homogenate was centrifuged at 100,000 × *g* for 30 min at 4 °C and the supernatant was collected as the cytosolic fraction. The remaining pellet was resuspended and incubated with buffer B (buffer A containing 1% Triton X-100) on ice for 20 min and then centrifuged at 100,000 × *g* for 30 min at 4 °C. This supernatant was collected as the membrane-associated fraction. Protein concentration was determined by Bradford dye-binding method (Bio-rad). Anti-PKCε antibody (1: 1000, Cell Signaling), anti-cleaved caspase 3 (1:1000, Cell Signaling) antibody were used to detect PKCε and cleaved caspase 3, respectively. Anti-GAPDH (1:10,000, Sigma) and anti-α1-sodium/potassium ATPase (1:1000, Santa Cruz) antibodies were used for housekeeping proteins. Uncropped images of the blots are presented in Supplementary Fig. 7.

### Immunohistochemistry

Anesthetized mice were perfused transcardially with PBS followed by 10% neutral buffered formalin. The brain and heart were dissected, fixed, and embedded in paraffin blocks. Five-μm sections were cut, mounted on the slide. Immunohistochemical staining of pERK was conducted using anti-pERK antibody (1:100, Cell Signaling). Cardiac sections were stained with hematoxylin and eosin (H&E) and picrosirius red to visualize the cardiac morphology and interstitial fibrosis^66^. Whole heart sections were scanned with Panoramic 250 FLASH II (3DHISTECH Ltd.). Percentage of fibrosis in the left ventricles was analyzed using ImageJ. For TUNEL assay, hearts were harvested 24 h after IR injury and embedded in OCT and then immersed in 2-methylbutane (2-ME) while 2-ME was chilled enough by liquid nitrogen. The heart sections (10 μm) were incubated with TUNEL reagents (Roche) according to the manufacturer’s instruction, and examined by fluorescence microscope. Whole heart sections were scanned and quantified using ImageXpress Micro Imaging XL system (Molecular Devices). TUNEL-positive signals were quantified using Cell Scoring algorithm of MetaXpress Software. This module detected and counted the TUNEL signal. We counted the TUNEL and DAPI signaling in the free wall of left ventricles from two sections (~60–100 μm apart) of each heart. These two sections were selected from the middle of each heart. The average total nuclei count for TUNEL assay is 45,663 ± 3804 (*n* = 6).

### Drug administration and viral infection

Cannulation was performed as described previously^36^. In brief, mice were anesthetized with vaporized isoflurane (1.5%) and their head were secured in the stereotaxic apparatus. A midline incision was made to expose the skull. A guide cannula was implanted above the target site. The stereotaxic coordinates for the intra-PVA infusion are 0 mm anteroposterior and 3.4 mm dorsoventral from bregma, and 26 gauge 9 mm stainless steel cannula, 33 gauge 9.5 mm infusion cannula were used. 0.3 μl of the drugs was injected over a period of 3 min using a syringe pump (KD Scientific), and the injection cannulae was left in place for an additional 2 min to allow the drug to diffuse. At the end of the experiment, brains were harvested and stained to verify the injection site.

PDBu, CNO, lidocaine, hexamethonium, propranolol, and glycopyrrolate were purchased from Sigma. U0126 and U0124 were purchased from Tocris. 0.4 mM PDBU, 10 mM U0126, and 10 mg/ml CNO were dissolved in 100% DMSO as stocks. Before applied to animals, PDBU and U0126 were diluted 1:1 in saline to a concentration of 0.2 and 5 mM, respectively. 50% DMSO were used for further dilution and as a vehicle control. CNO was diluted in saline to 0.5 mg/ml for applying to mice, and diluted in H_2_O to 1 mM for whole cell recording. Intra-PVA direction infusion of U0126 (1.5 nmol), U0124 (1.5 nmol), and PDBU or their vehicles (20 pmol) was performed at specific time points. IP injection of CNO (3 mg/kg) was performed 5 day after SNI surgery, myocardial IR injury was performed 3 h after CNO injection. Intravenously (i.v.) injection of hexamethonium (30 mg/kg), propranolol (2 mg/kg), and glycopyrrolate (0.4 mg/kg) was performed 15 min before myocardial IR injury. Intrathecally injection of lidocaine (15 mg/kg) was performed 5 day after SNI or sham surgery. Viral infection in PVA: adeno-associated virus (AAV) is obtained from UNC Vector Core, University of North Carolina School of Medicine. 0.3 μl of AAV-CaMKII-hChR2(H134R)-eYFP-EPRE (qPCR titer: 8.5 × 10^12^ vg/ml), AAV8-CamKIIa-hM4D-mcherry (Dot Blot titer: 3.3 × 10^12^ vg/ml), or AAV5-CaMKII-eYFP (qPCR titer: 6.3 × 10^12^ vg/ml) was infused into PVA. After 6–8 weeks, mice were subjected for the further examinations.

### Electrophysiology

Extracellular field potentials and the unit activities in PVA with sciatic nerve stimulation were conducted as previous description^67^. Mice were anesthetized with 1.5% isoflurane when the multichannel Michigan probe (NeuroNexus) was loaded to PVA at about 0.3 mm posterior and 0.7 mm lateral to bregma; probe inserted at the angle of 12° from the vertical line. An Ag–AgCl reference electrode was placed in the nasal cavity. The contact current was delivered by a cuffed electrode hooked on the left sciatic nerve (Model 2100, A-M System). The minimal effective pulse leads to toe prickling was defined as the stimulation threshold. The current strength from one to twenty fold of threshold was applied. After PVA spike unit activities were recorded, the optical fiber was loaded into D3V from the contralateral hemisphere of AAV-hChR2 infected mice at about 1 mm lateral, 3.2 mm ventral to bregma, and 15° from the vertical line. 5 pulse of 10 Hz blue light stimulation was applied. The sampling rate of recorded analog signals was 6 and 24 kHz for field potential and the unit data, respectively, and then the data were processed in a multichannel data acquisition system (TDT). The exact insertion site was identified by two electrical lesions. One of the sixteen contact points of the probe was amended as a lesion channel. A 10 s pulse of 25 μA current was applied after recording and the second lesion was made when the probe withdrawal 600 μm ascend to the first one.

Six to eight weeks after AAV infusion, mice were anesthetized with isoflurane (2%) followed by decapitation. The brain was quickly removed an immersed in an ice-cold artificial cerebrospinal fluid (ACSF) (in mM: 119 NaCl, 2.5 KCl, 26.2 NaHCO_3_, 1 NaH_2_PO_4_, 1.3 MgSO_4_, 11 glucose, and 2.5 CaCl_2_, the pH was adjusted to 7.4 by gassing with 5% CO_2_/95% O_2_). Whole brain was isolated and fixed by 3% agarose for slicing. Mice PVA slices were cut near bregma in thickness of 300 μm for the whole-cell recordings with a vibrating tissue slicer (D.S.K. Microslicer DTK-1000; Dosaka EM). Slices were allowed to recover at room temperature (24–25 °C) for 1.5 h in an interface-type holding chamber. The slices were transferred to an immersion-type recording chamber mounted on an upright microscope (Scientifica Ltd.) equipped with 40× water-immersion objectives. The oxygenated ACSF was continuously perfused at 1–2 ml/min. The patch pipettes pulled from borosilicate glass tubing (1.5 mm outer diameter, 0.86 mm inner diameter; G150F-4, Warner Instruments, resistance of 5–8 MΩ) and filled with internal solution which consisting of the following (in mM): 131 K-gluconate, 20 KCl, 10 HEPES, 2 EGTA, 8 NaCl, 2 ATP, 0.3 GTP, and 6.7 biocytin, with the pH adjusted to 7.2 by KOH and the osmolarity adjusted to 300–305 mOsm by distilled water. Recordings were made at room temperature (24–25 °C) with a patch amplifier (Multiclamp 700 B; Axon Instruments). For voltage-clamp recordings, the membrane potential of YFP-positive cell was held at −70 mV, a series of blue light pulses were applied to elicit inward currents. Then the mode shifted to current clamp recording to record the evoked action potential. Evoked spontaneous action potentials were recorded before and after 10 μM CNO perfusion. Data were discarded when the Rs varied by >20% from its original value during the recording. All signals were low-pass filtered at a corner frequency of 1 kHz and digitized at 10 kHz using a Micro 1401 interface (Cambridge Electronic Design). Data were collected using Signal software (Cambridge Electronic Design). The blue light source was OptoLED Light source system (CAIRN Research Ltd.). Light pulses of 10 ms 5 Hz and intensity 5 mW mm^−2^ were used.

### Optical stimulation

Six to eight weeks after AAV infusion, mice were implanted with 23 gauges, 9.5 mm stainless steal cannula above the PVA brain region before optical stimulation. Mice were singly housed after implantation and recovered for at least 7 days before behavior tests. Mice that received both optical stimulation and IR injury were anesthetized with isoflurane (2%) before optical stimulation. The optical fiber (UM22-200–0.22 NA, Ø200 µm Core; Thorlabs) was directly inserted into the cannula and connected to rotary Joint direct linked to LED driver (Doric Lenses). Blue light was delivered in 10 Hz, 10 ms pulse width, 2.8 mW mm^−2^. Ten minutes after optical stimulation, the mice were then subjected to IR injury procedure.

### Telemetry measurement of electrocardiography

ECG was measured using miniature telemetry implant (model: HD-X11, DataSciences International). The telemetry probe was implanted according to the manufacturer’s instruction. The animal was recovered for 1 week before measurement of ECG. Each mouse was habituated for 10 min in their individual cage before telemetry recording. For the SNI group, the mice underwent ECG recording for 2 h before and 5 days after SNI surgery. For the optical stimulation group, after insertion of the optical fiber, the ECG was recorded for 30 min each before and during blue light stimulation. The ECG data were analyzed using LabChart 7 (AD Instruments). Six stable 1 min ECG recordings were chosen for the analysis of heart rate.

### Vagus nerve stimulation

Mice were habituated in the operation room for at least 2 h before subjected to EKG recording (ML870, Powerlab 8/30, ML136, Animal Bio Amplifier, ML221, Bridge Amplifier, and MLS023, Chart Software, AD Instruments) under anesthetization (1.5% isoflurane). After a 10-min EKG recording, an incision on the neck was made to expose the left external jugular vein, carotid artery, and vagus nerve. Then the left vagus nerve was gently isolated without damaging the vessels and crushing the nerve. The electrical stimulation is adapted from the sciatic nerve stimulation^67^. In brief, the contact current was delivered by the silicon cuffed Teflon-coated silver electrode hooked on the left vagus nerve (Model 2100, A-M System; MyoPacer Field stimulator, IONOPTIX, Corp.). Inside the cuff, the silver exposure sites attached directly to the nerve which surrounded by mineral oil as the insulate sheet. To be consistent with the optogenetic stimulation, a 10 Hz, 7–10 μA current was applied to the left vagus nerve for 5 min. During this procedure, the heart rate was monitored by EKG recording. An effective stimulation led to a 20–30% of heart rate drop and a rebound of heart rate was observed as the stimulation stopped. After vagal stimulation, the mouse was then subjected to IR surgery immediately. Any inappropriate stimulation or operation led to the nerve damage or ineffective heart rate alteration was excluded. A 0 μA current was applied to nerve as the sham vagal stimulation.

### Serum creatine kinase-muscle and brain isoenzyme measurement

One day after IR injury, blood were taken from right ventricle before mice were sacrificed and allowed to clot by leaving it for 15 min at room temperature. Serum was collected after centrifugation (2000 × *g* for 10 min at 4 °C). Serum level of CK-MB was measured by Fuji Dry-chem 3500s analyzer (Fuji Film), according to the manufacturer’s instructions.

### Echocardiography and blood pressure measurement

An ultrasound unit (IE33, Philips) with a 15–7 MHz linear transducer was used. The animals were anesthetized using avertin (25 mg/10 g, IP. Sigma) for echocardiographic examination. After anesthesia the animal was shaved on the chest wall, and sono-gel was used. Under M-mode sonography the diameter of aortic outflow track and the size of left atrium were measured in long axis view of heart. Short axis M-mode at the level of the papillary muscle was used for measurement of diastolic and systolic diameter of LV, as well as the wall thickness. Fractional shortening was calculated by using a standard formula. All parameters were measured three times and their averages were recorded.

Non-invasive blood pressures were measured using blood pressure analysis system (BP-2000, Visitech Systems) in Taiwan Mouse Clinic.

### Data analysis

The number of samples (*n*) was shown in the text and figures. All the experiments have been replicated at least three times in the laboratory. The sample size not was predetermined. All the data were presented as mean ± SEM. Mechanical withdrawal ratios were analyzed by two-way repeated measures ANOVA, followed by Holm–Sidak post hoc analysis. One-way ANOVA with multiple comparisons (Figs. 1, 4, 7) and Student’s *t*-test was used for comparison of two groups. All the experiments data passed the normality test (Shapiro-Wilk), except the experiments using propranolol and TUNEL, FS and EF of echocardiography in SNI/sham 5 days after SNI, fibrosis in sham receiving U0124/U0126, IR injury 4 weeks results of echo in EF and LVIDs in SNI/sham and LVPWd in ChR2/eYFP and quantification of ERK phosphorylation in immunostaining. Kruskal–Wallis one-way analysis of variance on ranks was used for these sets of data. We did not estimate variations in the data. The variances are similar between the groups that are being statistically compared. A value of *p* < 0.05 was regarded as significant. Heart rate results were compared in the ChR2-infected group by paired *t*-test.

### Data availability

All relevant data are available from the authors upon request.

## Electronic supplementary material

Supplementary information
