# Author Correction: Cardioprotection induced in a mouse model of neuropathic pain via anterior nucleus of paraventricular thalamus

**DOI:** 10.1038/s41467-018-04160-5

**Published:** 2018-04-23

**Authors:** Yi-Fen Cheng, Ya-Ting Chang, Wei-Hsin Chen, Hsi-Chien Shih, Yen-Hui Chen, Bai-Chuang Shyu, Chien-Chang Chen

**Affiliations:** 1 Graduate Institute of Life Sciences, National Defense Medical Center, Taipei, 114 Taiwan; 2 Institute of Biomedical Sciences, Academia Sinica, Taipei, 115 Taiwan; 3 International Graduate Program in Molecular Medicine, National Yang-Ming University and Academia Sinica, Taipei, 115 Taiwan; 4 Molecular Medicine Program, Taiwan International Graduate Program, Institute of Biomedical Sciences, Academia Sinica, Taipei, 115 Taiwan

Correction to: *Nature Communications* 10.1038/s41467-017-00891-z, published online 10 October 2017

The original version of this Article contained an error in the affiliation of the second author, Ya-Ting Chang. The correct affiliations for Ya-Ting Chang are Institute of Biomedical Sciences, Academia Sinica, Taipei 115, Taiwan and International Graduate Program in Molecular Medicine, National Yang-Ming University and Academia Sinica, Taipei 115, Taiwan.

