# Supplementary material for: Cardioprotection induced in a mouse model of neuropathic pain via anterior nucleus of paraventricular thalamus
Source: Nat Commun. 2017 Oct 10;8:826. doi: 10.1038/s41467-017-00891-z (PMC5635036; doi:10.1038/s41467-017-00891-z)

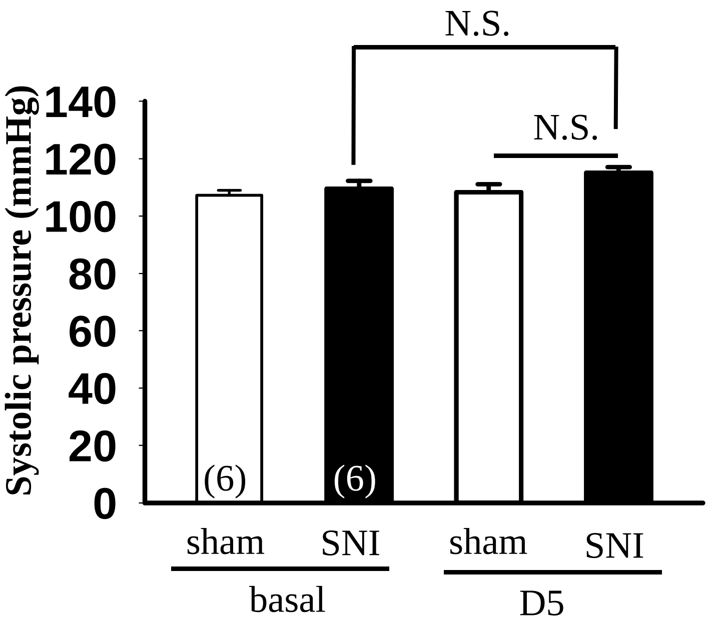

Figure S1. Systolic blood pressure before and after sham/SNI surgery. There is no significant difference in the systolic blood pressure between the sham and SNI groups.

Echocardiographic analysis

	LVIDd (mm)	LVIDs (mm)	LVPWd (mm)	LVPWs (mm)	EF (%)	FS (%)
**Sham 5d (5)**	3.7±0.21	2.65±0.13	0.64±0.04	0.97±0.04	68.03±2.66	32.58±2.05
**SNI 5d (5)**	3.81±0.14	2.65±0.2	0.66±0.05	0.97±0.04	64.72±4.42	31.00±3
**sham IR4w (5)**	4.36±0.20	3.16±0.18	0.75±0.03	1.14±0.10	60.12±4.38	27.86±2.71
**SNI IR4w (5)**	3.85±0.16	2.43±0.14*	0.79±0.03	1.24±0.10	74.3±1.35*	37.48±1.25*
**sham IR4w/ U0124 (5)**	4.52±0.31	3.64±0.36	0.76±0.05	1.00±0.04	45.6±5.20	19.4±2.58
**sham IR4w/ U0126 (5)**	4.20±0.13	3.18±0.12	0.72±0.06	0.96±0.07	55.60±1.44	24.80±0.86
**eYFP IR4w (5)**	4.42±0.16	3.46±0.21	0.78±0.04	1.04±0.04	49.40±4.00	21.60±2.13
**ChR2 IR4w (5)**	3.90±0.15	2.35±0.17#	0.76±0.05	1.33±0.18	73.48±3.46#	36.94±2.39#

Figure S2. Parameters of echocardiographic measurement.

LVIDd, left ventricular internal dimension at end diastole; LVIDs, left ventricular internal dimension at end systole; LVPWd, left ventricular posterior wall at end diastole; LVPWs, left ventricular posterior wall at end systole; EF ejection fraction; FS, fraction shortening. The number in the parenthesis is sample size. * p<0.05, compared to Sham IR 4w group. #p<0.05, compared to eYFP IR group.

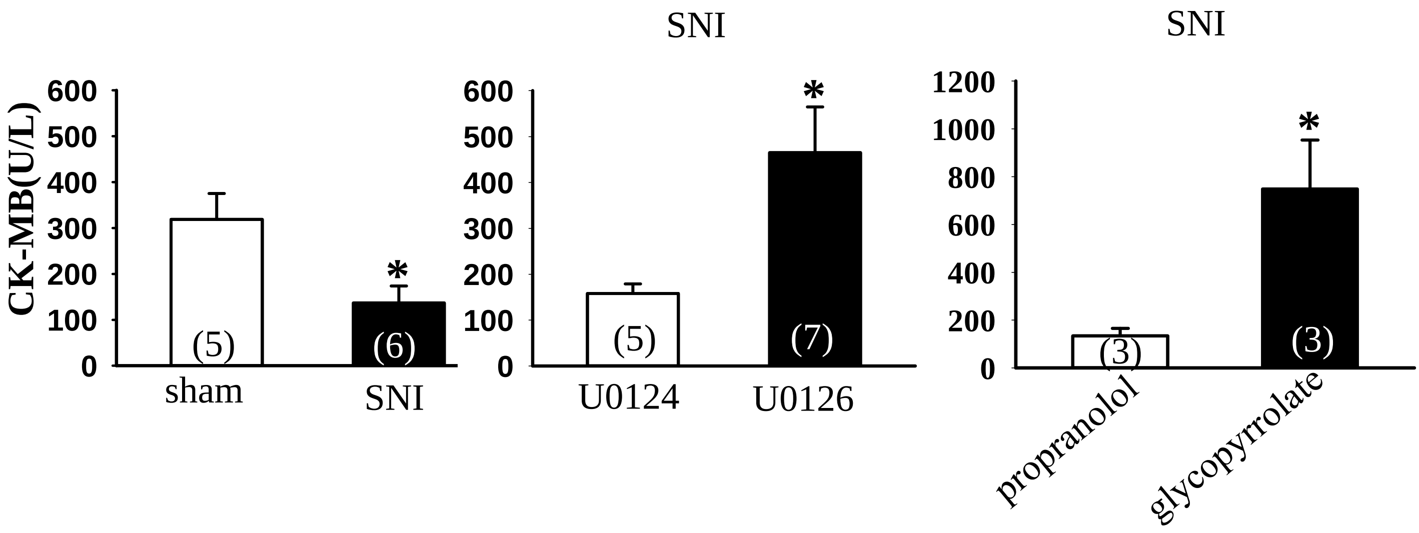

Figure S3. Serum CK-MB measured one day after IR reperfusion. Serum CK-MB level was significantly higher in sham group compared to SNI group, in U0124 treated group compared to U0126 group, in propranolol group compared to glycopyrrolate (*p<0.05). These results support our idea that neuropathic pain provides cardiac protection after ischemia reperfusion.

Supplementary Figure 4

Cheng et al.

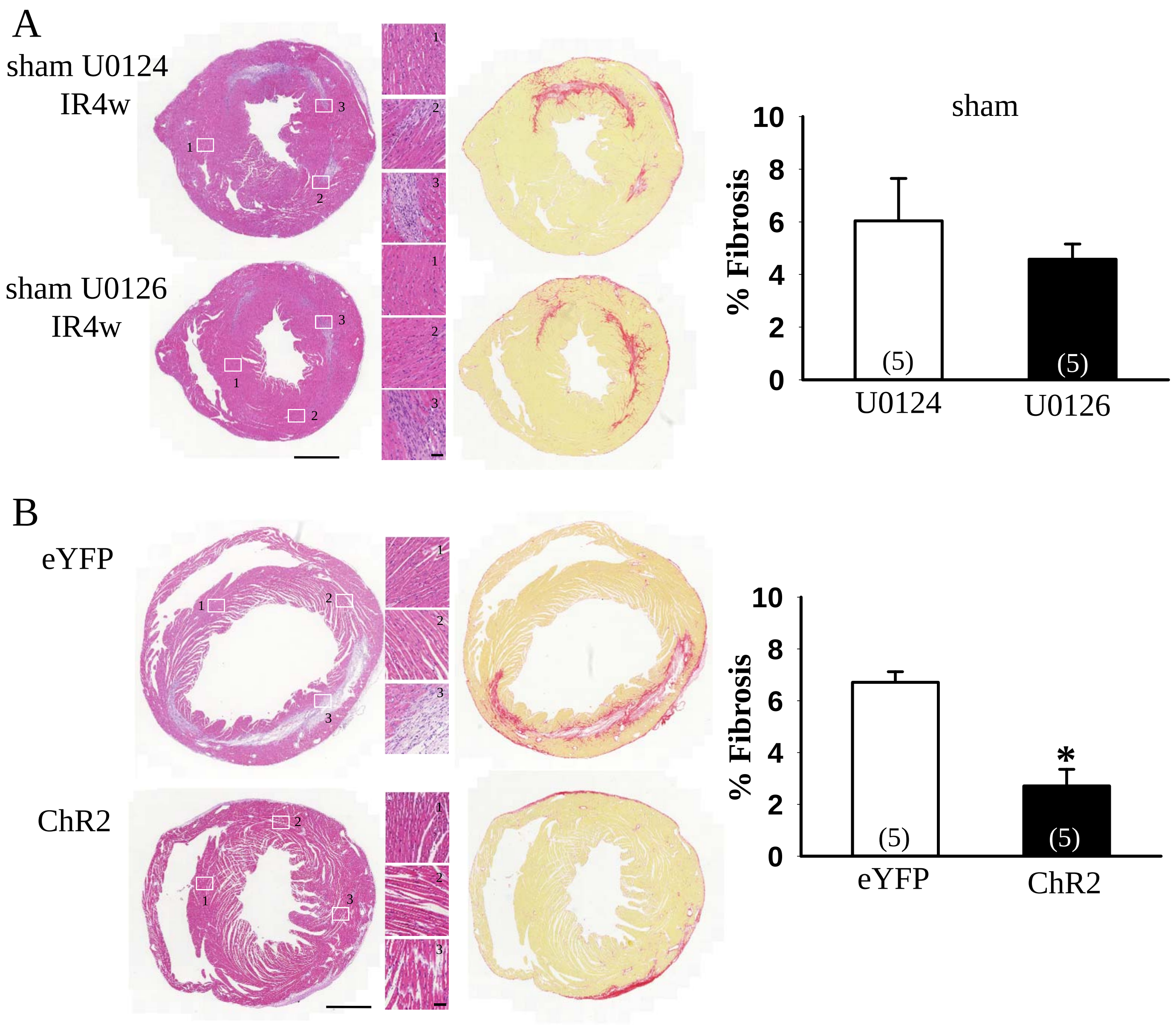

Figure S4. Representative images of H&E staining (left panel) and picro-sirius red staining (right panel) of cardiac sections 4 weeks after IR injury. Areas marked by white rectangles were magnified and shown in the middle panel. (A) Images of cardiac sections from sham animals treated with U0126 or U0124. Bar graph, quantification results of fibrosis in left ventricles. (B) Images of cardiac sections from eYFP or Ch2R animals received blue light stimulation. Bar graph, quantification results of fibrosis in left ventricles. *p<0.05, compared to eYFP group. Scale bar=1 mm and 50 μm for the lower and higher magnified images, respectively.

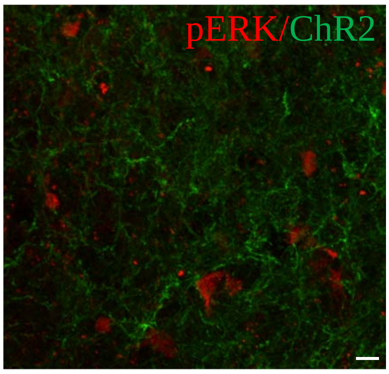

Figure S5. Repetitive optogenetic stimulation of PVA neurons increased pERK staining in PVA. Scale bar, 10 μm.

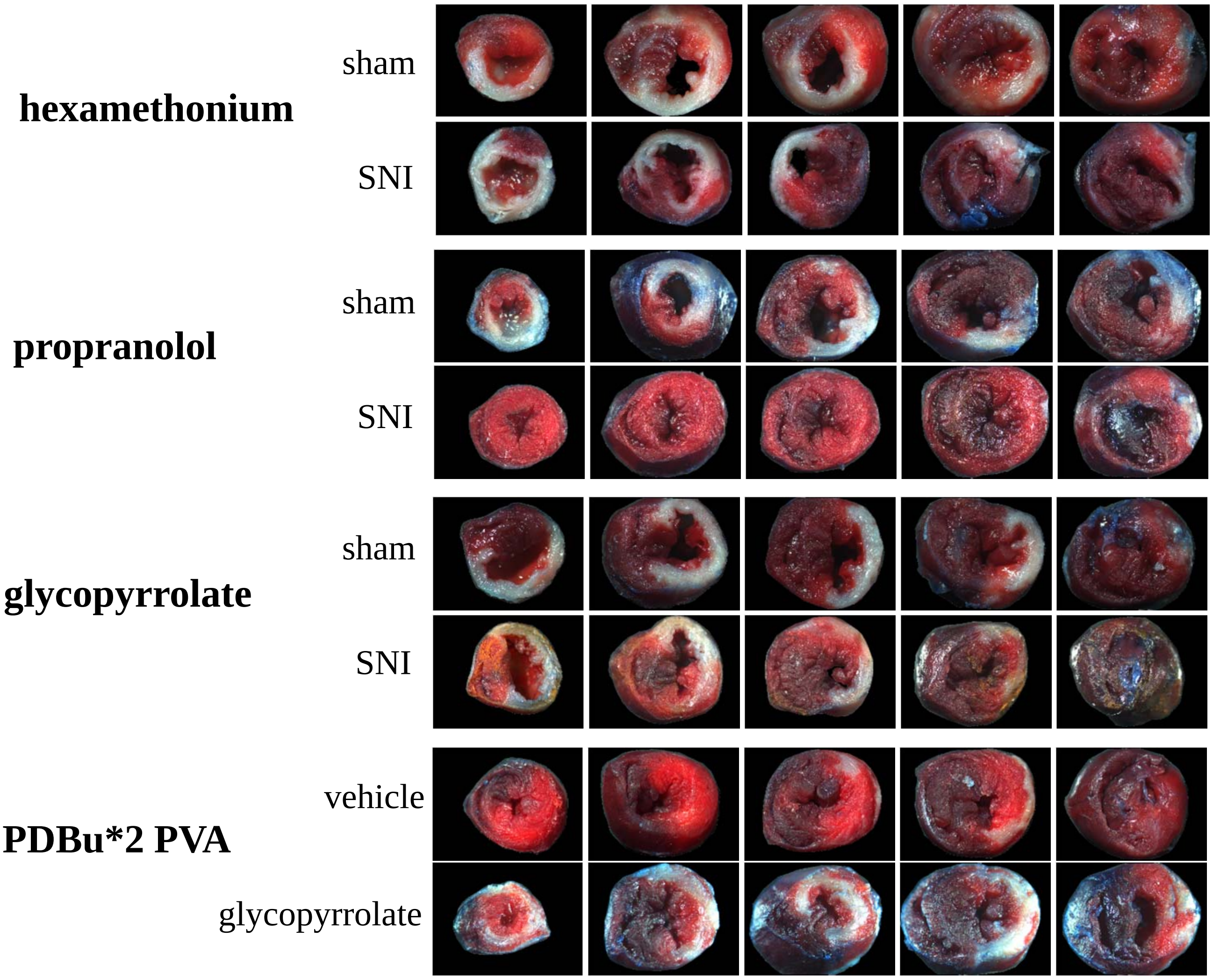

Figure S6. Complete serious of TTC stained cardiac section shown in Figure 8.

Supplementary Figure 7

Figure 1E

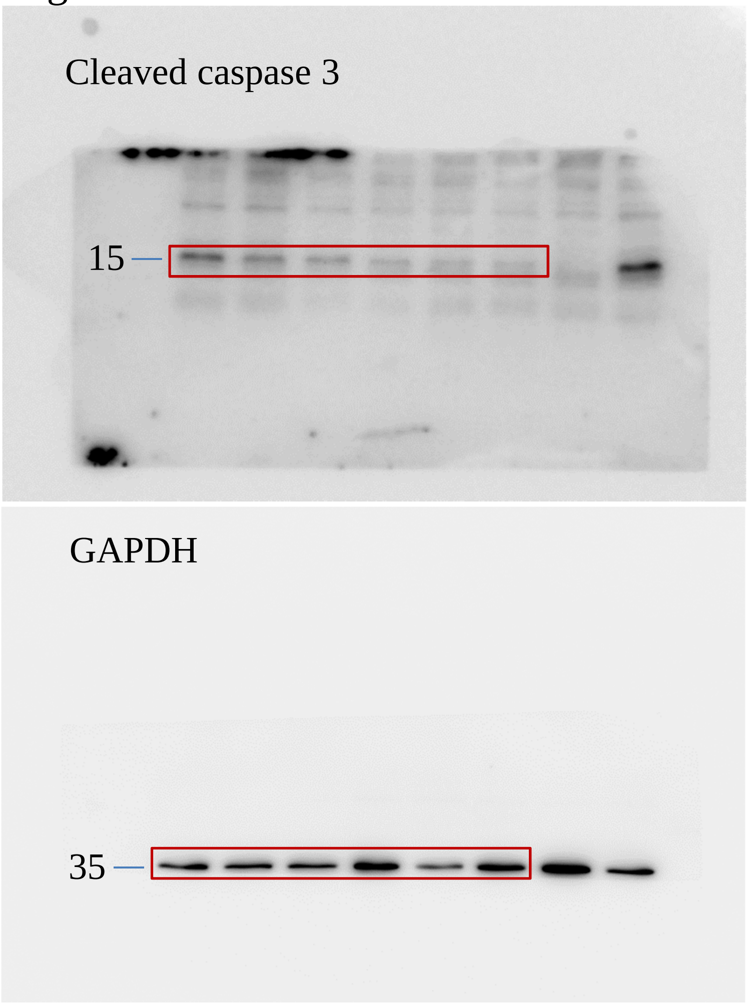

Figure 1G

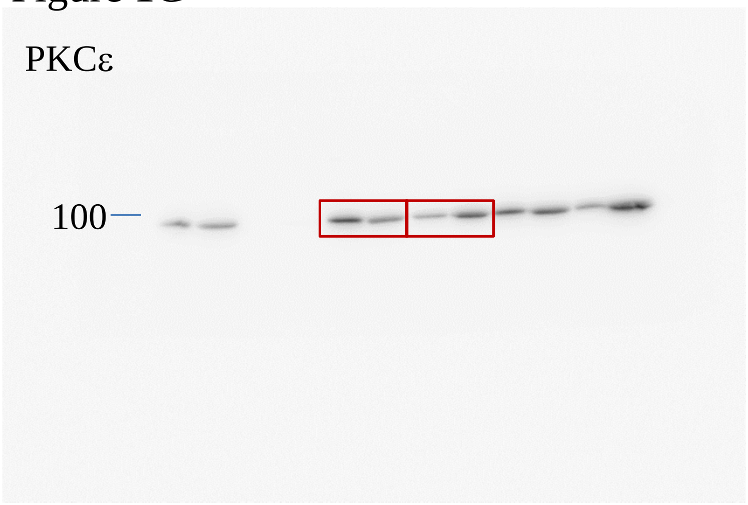

Supplement: Supplementary file 1 — Supplementary information [file 41467_2017_891_MOESM1_ESM.pdf]
